# Books Average Previous Decade of Economic Misery

**DOI:** 10.1371/journal.pone.0083147

**Published:** 2014-01-08

**Authors:** R. Alexander Bentley, Alberto Acerbi, Paul Ormerod, Vasileios Lampos

**Affiliations:** 1 Department of Archaeology & Anthropology, University of Bristol, Bristol, United Kingdom; 2 Volterra Partners LLP, London, United Kingdom; 3 Anthropology Dept, Durham University, Durham, United Kingdom; 4 Department of Computer Science, University of Sheffield, Sheffield, United Kingdom; University of Maribor, Slovenia

## Abstract

For the 20^th^ century since the Depression, we find a strong correlation between a ‘literary misery index’ derived from English language books and a moving average of the previous decade of the annual U.S. economic misery index, which is the sum of inflation and unemployment rates. We find a peak in the goodness of fit at 11 years for the moving average. The fit between the two misery indices holds when using different techniques to measure the literary misery index, and this fit is significantly better than other possible correlations with different emotion indices. To check the robustness of the results, we also analysed books written in German language and obtained very similar correlations with the German economic misery index. The results suggest that millions of books published every year average the authors' shared economic experiences over the past decade.

## Introduction

Whereas it is self-evident that individuals must rely on past observations to anticipate the consequences of future decisions [1]–[3], the time scale over which observations are aggregated, particularly at the population level, is a pertinent evolutionary question. A characteristic time-scale of about 30 years in the usage of new words, for example, may be a generational effect within recent centuries of language evolution [4]. This may be a relatively modern effect, however, and now the instantaneous nature of the Internet contrasts even more with millennia of cultural evolution that allowed humans to accumulate information and learn skills over many generations [5]–[9].

Over a much longer time scale, written language not only accumulates technological knowledge but it continually regenerates the cultural basis by which people make sense of their own experience and maintain their social relationships [10], [11]. The weighting of this experiential knowledge can be affected through the expression of associated emotions [10], [12]–[16].

We hypothesize that book authors, as both producers and consumers of shared knowledge, are informed by past economic conditions at some definable time scale – they convey common knowledge not just factually but also indirectly, through emotional content. Accordingly, we would expect to find a positive correlation between mood, as expressed in books, and economic conditions in the recent past. As books take time to write and publish, we expect that each year of publication may average past economic influences over a definable number of years.

The relationship between emotions and current events can be explored through the increasing availability of recorded language use at the population level [17]–[24]. Language expressing emotion has recently been correlated with socio-political and economic trends [25]–[28], amid a wealth of studies exploring how large samples of online language use can be statistically predictive future consumer activity, unemployment rates, concerns about personal health and other collective behavior [29]–[35]. A “forward looking” index using Google trend data that shows strong correlation with GDP per capita further evidences the connection between crowd-sourced textual data and broad economic indicators [36].

To complement the vast study of social media on the scale of days or hours, we explore whether human sentiments, aggregated by language use at the population scale, reflect not just with specific historical events but with more general economic conditions of the past. Focussing on the 20^th^ century, through the Google Books Ngram corpus [4], [37]–[39], we examine how well a new index of emotions in books [40], or literary ‘misery index’, 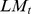, correlates with the widely-used economic ‘misery index’, 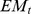, which is the unemployment rate plus the inflation rate [41], [42].

Our simple model is that the literary misery index will be proportional to the moving average of economic misery, 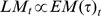, where *τ* denotes the period over which we take the moving average of the annual U.S. misery index scores (years leading up to and including the current year). The moving average is based solely on the past, in that it is a non-weighted arithmetic mean of the past 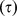 years of economic misery.

As described in the Methods section below, we used the WordNet Affect (WNA) text analysis tool [43]–[45] to extract literary mood from the Ngram corpus [40]. We also compared our results with two independent emotion extraction tools, Linguistic Inquiry and Word Count [46] and a recently proposed “hedonometer” method [47].

## Results

**Figure 1:**
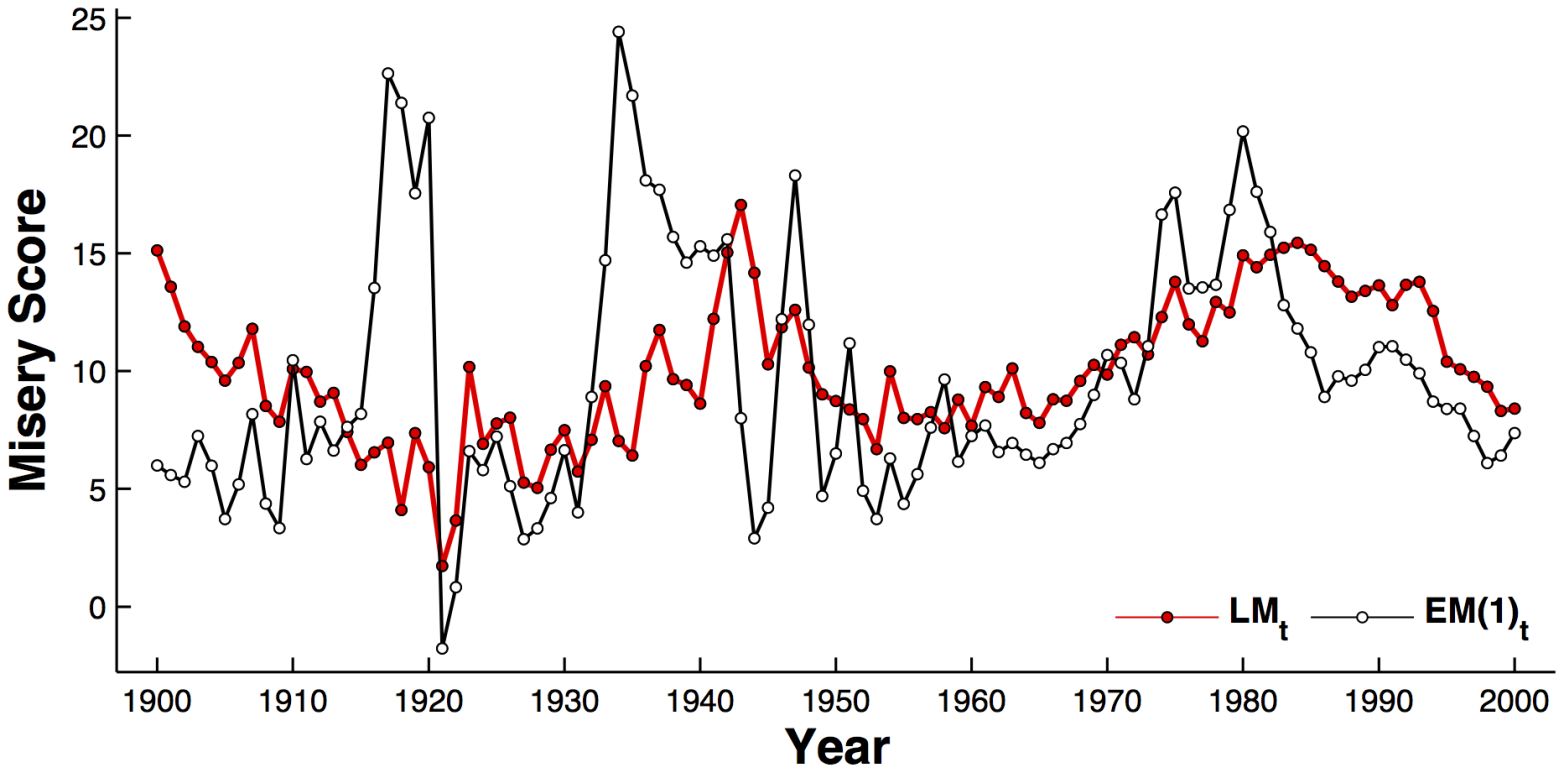
Time series of the literary misery index 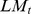 for all books calculated through WNA (white circles), versus the U.S. economic misery index 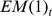 (red circles). 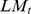
 has been scaled by a factor of 10 to allow a better comparison.

Figure 1 compares the time series of the literary misery index, 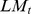, derived from the WNA sampling of all English books (see Methods), versus the contemporaneous U.S. economic misery index 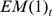. There is some correlation (Pearson's 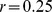) even when we do not take any moving average of the economic misery index, 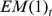. Visually, the literary misery index seems to respond to major phases of the 20^th^ century: literary misery increased after the economic Depression, then declined after the post-War years, then rose again after the recession of the 1970s, and declined again following on from the economics recovery of the late 1980s. The literary misery time series is well-characterised (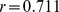) by a sine wave with a period of 41.6 years, phased such that a 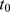 year would be 1890. Spectral analysis also indicates a similar periodicity in the economic misery index, though this is not as clearly defined, being of the order of 25–50 years. Importantly, this frequency range is far more important than the period of 5–12 years which is widely believed to characterise the economic business cycle of the fluctuation in real GDP growth.

**Figure 2:**
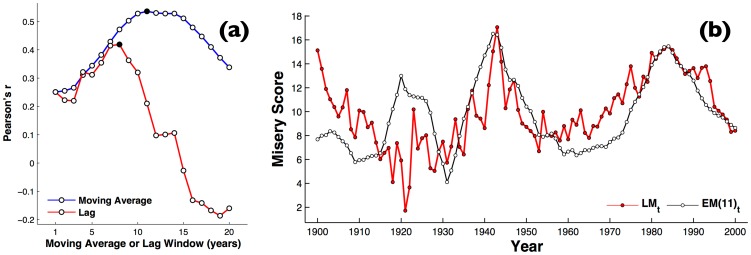
Moving average of economic misery. (a) The effect of varying the moving average period, *τ*, versus a simple lag, on correlation expressed as Pearson's *r* between the time series of 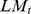 for all books of 1900–2000 and U.S. misery index (b) Time series for 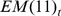, the 11-year moving average of the U.S. misery index (red circles), versus literary misery index, 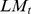, derived from all books calculated through WNA (white circles). Similarly to Figure 1, 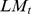 has been scaled by a factor of 10 to allow a better comparison.

When we compare the correlations between 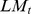 and all the values of moving average (*τ* up to 25 years) for the economic misery index (see Figure 2a), we find a peak in the overall goodness of fit at 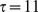 (Pearson's 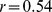). The moving average correlates significantly better than the best fit using a simple lag, which is 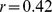 at a lag of 7 years, i.e. between 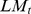 and economic misery 7 years previous. Figure 2b shows the time series for 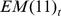, the 11-year moving average of the U.S. misery index, versus literary misery index, 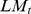, derived from all books calculated through WNA. Notice that, as shown in Figure 1, the volatility of the economic conditions has been larger than have been the changes in literary misery, so 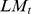 has been scaled by a factor of 10 to allow a better comparison.

**Figure 3:**
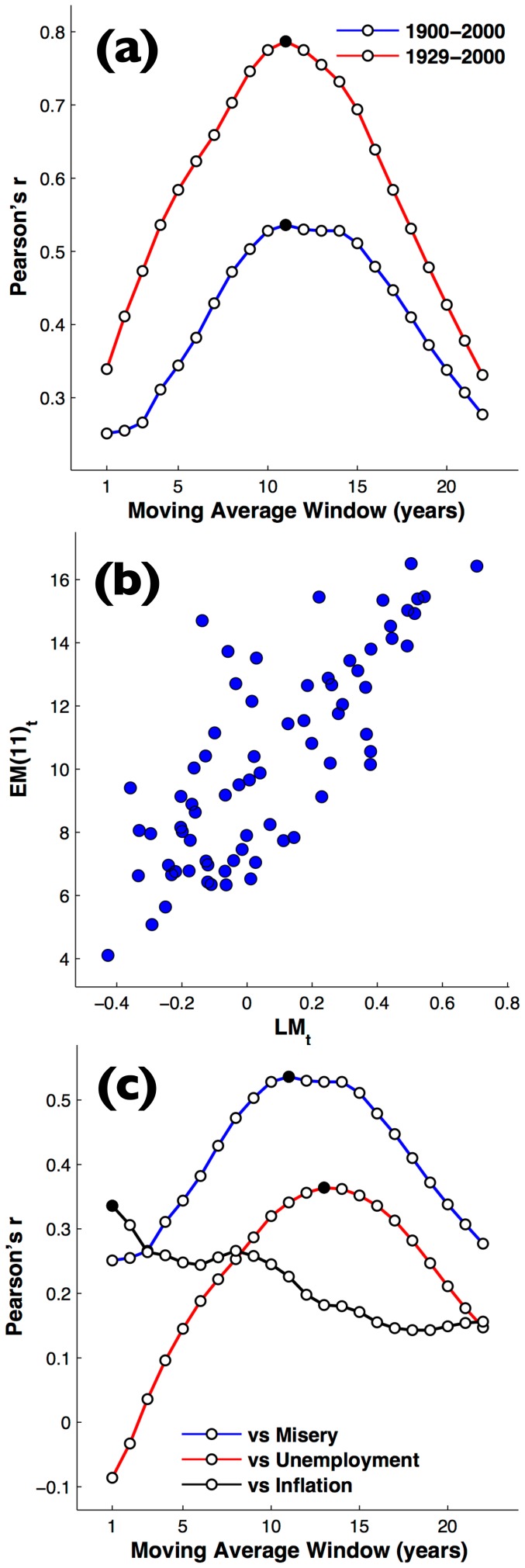
Time period and economic index. (a) The effect of varying the moving average period, *τ*, on correlation expressed as Pearson's *r* between the time series of 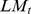 for all books in the Google data and U.S. economy misery index, 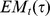 from 1900 to 2000 (blue line) and between the the two same time series from 1929 to 2000 (red line) (b) Scatterplot of literary misery index, 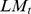, versus the 11-year moving average of U.S. economy misery index, 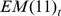, from 1929 to 2000. (c) The effect of varying the moving average period, *τ*, on correlation expressed as Pearsons *r* between the time series of 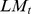 for all books in the Google data of 1900–2000 versus U.S. misery index (blue line), U.S. unemployment rate (red line), and U.S. inflation rate (black line).

A closer relationship between 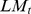 and 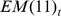 distinctly begins with the economic Depression following the 1929 crash (Figure 2b). When we compare the two time series considering only years from 1929 to 2000, we find again a peak in the overall goodness of fit at 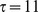 years (Pearson's 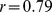), and correlations are overall considerably better than for the entire 20^th^ century, for all values of moving average *τ* up to 22 years (Figure 3a), and still significantly better than a lag (the lag peaks at 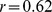 for a lag of 8 years). The scatterplot in Figure 3b shows how the literary misery score, 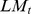, correlates with the 11-year moving average of yearly U.S. Misery index, 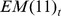, from 1929 to 2000.

It could be argued that the results are primarily due to three data points. Economic misery coincides with WW1 (1918), the aftermath of the Great Depression (1935) and the energy crisis (1975). We addressed this point directly by calculating the correlations on a ‘leave-one-out’ basis. In other words, when a correlation is calculated using data from 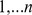, leave-one-out calculate the correlation over the periods (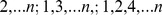, etc). The results are therefore robust, and do not depend upon the potential ‘extreme’ data points.

The economic misery index, 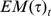, correlates better with the literary misery index than do the individual components of the economic misery index – unemployment or inflation – on their own. As shown in Figure 3c, these correlations with U.S. misery index, 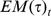, are better than the correlations versus U.S. inflation (best 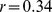) or U.S. unemployment (best 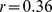). Note that while both correlate positively with literary misery, U.S. unemployment and U.S. inflation themselves were moderately anti-correlated during the 20^th^ century (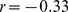 for whole century and 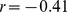 from 1929 to 2000), such that their sum, economic misery, best correlates with literary misery via the moving average. Note that the U.S. and U.K. economy misery indices were themselves correlated from 1929 to 2000 (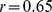).

Table 1 shows the statistically significant correlations between 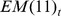 and literary misery indices calculated in other subsets of the English corpora, or in the same main corpus (all Books in English) but with other techniques (see Methods). We also calculated correlations between 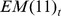 and WNA and LIWC single moods. Interestingly, all the best correlations involve our ‘composite’ literary misery index (as opposed to single moods). Two independent ways to calculate the literary misery index (LIWC and Hedonometer) give positive and significative correlations with 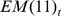. Comparing all values of moving average for all English books up to 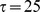 years, the same value of 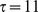, which yielded the best correlation for WNA, also gives the best correlation both for LIWC and Hedonometer Table 1), while the very best correlations for British books (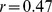) or American books (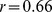) alone are at 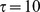.

**Table 1:** Correlations between the 11-year moving average on the U.S. misery index and different literary samples, 1929 to 2000.

Literary index	Pearson's	*p* (<) at	std. dev.	95% conf.
	*r*	that *r*	on *r*	interval on *r*
WNA misery (all English books)	0.79	0.0005	0.05	0.67–0.87
WNA misery (American books)	0.65	0.0005	0.07	0.51–0.76
LIWC misery	0.55	0.0005	0.08	0.37–0.70
WNA misery (British books)	0.46	0.0005	0.09	0.27–0.62
Hedonometer misery	0.31	0.01	0.12	0.00–0.49
LIWC negative emotions	0.27	0.05	0.11	0.03–0.47
WNA disgust (all English books)	−0.25	0.05	0.13	−0.49–0.17
LIWC sadness	0.23	0.05	0.13	−0.01–0.50

Highest magnitude correlations at top. Standard deviations and confidence intervals on 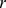 are estimated by 10,000 bootstraps (random sampling with replacement). Not shown are the literary scores which did not yield statistically significant correlations with economic misery: WNA misery (Fiction books), WNA joy, WNA fear, WNA surprise, WNA anger, WNA sadness, LIWC anxiety, LIWC anger, LIWC affect, and LIWC positive emotions.

These results are robust, but to explore other possibilities we repeated the analyses on a composite index in which we simply averaged the U.S. and British misery indices for each year, and again found that the best correlation (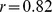) with WNA ‘misery’ extracted from All English Books occurred at a moving average of 11 years, and that the best correlation of the averaged UK/US economic misery with LIWC misery (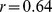) occurred at 10 years moving average.

**Figure 4:**
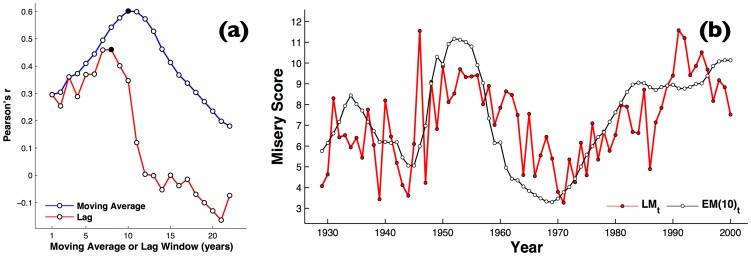
Repeating the analysis on German economic and literary misery. (a) The effect of varying the moving average period, 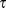, versus as a simple lag, on correlation between for German LIWC and German misery index (b) Time series of the literary misery index for all books calculated through German LIWC (white circles), versus the German economic misery index (red circles).

Finally, to check these patterns against a non-English language, we analysed the correlations of a literary misery index derived from LIWC sampling of German books, with the German EM index from 1929–2000. We get an optimal Pearson correlation of 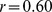 for a window of 10 years (Figure 4).

## Discussion

We have found a distinct positive correlation between a literary misery index in English (and German) language books and a moving average of the economical misery index. It is a common observation that culture cycles [20], [48]–[50], and given that both literary misery and economic misery follow cycles, it could be counter-argued that the correlation here has no causal link.

We think it is highly unlikely that there would be no causal relationship, for a few reasons. Firstly, the best correlation is achieved when we match literary misery with economic misery specifically, rather than different emotions compared to economic misery, or literary misery to unemployment or inflation individually. This implies a causal connection; it actually matters that we match the emotional index to the economic index.

Secondly, the moving average of economic misery correlates better with literary misery than economic misery after a specific number of years time lag. The results support the hypothesis that emotion words in books reflect general past economic conditions averaged over a time scale of approximately the previous decade. This underlies the logic of causal connection we are proposing, which is that populations accumulate experience over a period of time, rather than referring back to a period of some specific number of years ago, via a lag.

Finally, there is sociological reality that unemployment, one of the two components of economic misery, generally has a negative effect on emotional state [51]–[53]. Unemployed workers are more likely to experience depression, anxiety, and poor self-esteem [54] and even increased mortality rates related to alcohol and smoking [55], [56]. This affects not just individuals, but also families and communities [57], [58].

At the population scale, it seems to take about a decade for these effects to become registered in books. Using different techniques for calculating the literary misery index on multiple corpuses, we find agreement for the best period of 10–11 years for the moving average. This is somewhat shorter than the generational time scale observed in the usage of new words [4] or in the collective drift in ‘content–free’ words tied to group style and identity [59]. Underlying this difference may be the event-driven nature of economics, injecting a continual stream of novelty into cultural evolution that is perhaps more subject to the limits of individual memory retrieval [1], [60].

Perhaps this ‘decade effect’ reflects the gap between childhood, when strong memories are formed, and early adulthood, when authors may begin writing books. Consider, for example, the dramatic increase of literary misery in the 1940s, which follows the Great Depression of the 1930s. As documented by a long term study of 167 Californians born in 1920–1921, the Depression Era left long-lasting impressions on their memories [61]. Authors from this generation would have begun writing in the 1940s.

In fact, the most significant correlation between emotions in books and past economics begins with the year 1929, persisting for the remainder of the 20^th^ century. This suggests that the global Depression instigated a more direct relationship between books and past economics. New urbanisation, mass production, and advertising in the 1920s appear to have effected a lasting change in emotional expression in literature [62]. The subsequent increase in ‘narcissistic’ word usage [26], [28] may reflect a growing 20^th^ century trend to interpret global economic events on a personal emotional level.

Written language enables the accumulation of factual knowledge, an evolutionary process as old as Palaeolithic cave art [8], [63]. In smaller societies, shared emotional knowledge provides an incentive and means for humans to cooperate [64]. One of these means is indirect speech, which incorporates shared knowledge of other people's feelings, and helps preserve social relationships by providing ways of ‘saving face’ amid continual complex negotiations of cooperation [11]. Joking relationships, for example, which require shared emotional and kinship knowledge, are essential for the cohesiveness of small traditional societies [65].

These relationships are part of the unique dynamics of cultural niche-construction, as humans must adapt to a ‘cognitive niche’ of knowledge-using and socially interdependent individuals, among whom “cognitive schemas and social emotions that evolved for one domain can be pressed into service for another and assembled into increasingly complex mental structures,” [64]. During the 20^th^ century, this cognitive niche began to include global economics as part of the shared emotional experience of populations.

It is certain that more literary cycles of this sort and their correlations with world events will be discovered; showing that in addition to predicting the near future using very recent event data [25], big-data can also be used to understand cultural dynamics on much longer time scales [23], [26], [38], [39], [59].

## Methods

We extracted literary mood from the Ngram corpus using lists of semantically related terms provided in WordNet Affect [43]–[45], and we additionally validated this method with two alternative and independent tools, the Linguistic Inquiry and Word Count [46], and the recent “hedonometer” method [47], originally proposed to analyse Twitter data.

We obtained the time series of words frequencies from the Google Books Ngram corpus using Version 2 (released in July 2012). Version 2 includes more than 8 millions books (versus the about 5 millions of the previous version) and better OCR results [37]. We considered English-language books, in four distinct corpora (all books in English, fiction books in English, American English books, and British English books).

We used the WordNet Affect (henceforth WNA) text analysis tool which groups synonymous terms into lists related to mood states to perform text analysis on these words after they had been stemmed using Porter's Algorithm [66]. This method is consistent with numerous other text-mining studies such as [22], [67]. We considered six distinct main emotions: anger (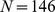), disgust (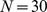), fear (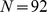), joy (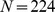), sadness (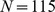), and surprise (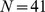).

For each stemmed word we collected the amount of 1-gram occurrences (case insensitive) in each year from 1900 to 2000 (both included). Following [38], we excluded data from years after 2000 because books published recently are still being included in the data set, and therefore latest records are incomplete and possibly biased. We normalized the frequency of the words in these word lists, then computed the average normalized frequency per year:
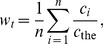
(1)where 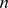 is the number of words in the list, 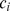 is the word count for word 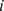 in the list in year 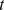, normalized by 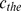, which is the count of the most frequent English word, ‘the’, in year 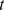. We normalized the yearly amount of occurrences using the occurrences, for each year, of the word ‘the’, rather than by the annual total number of words scanned, to avoid the effect of the influx of data, special characters, etc. that may have changed considerably over the 20^th^ century [40], [68].

These normalized frequency scores were then converted to their 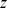-score equivalents as
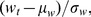
(2)where 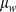 and 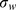 are the mean and standard deviation of 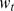 over the 100 years of the 20^th^ century. We denote the 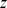-score equivalents in year 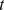 for the 224 listed ‘joy’ words and the 115 listed ‘sadness’ words as 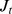 and 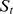, respectively, and the difference between them, 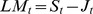 as our literary misery index, 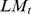.

We note that normalising by ‘the’ is not a significantly determinant of our results. Over the twentieth century, frequencies of ‘the’ exhibit none of the cycling patterns of the emotion words. To confirm that this was not a factor, we re-calculated the literary misery index from All Words, but normalised this time with the yearly frequencies of the word, ‘of’ rather than ‘the’, in the denominator of eq.(1). Using these re-normalised values of 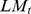 we find virtually identical results to those presented in our Results section: a peak in the overall goodness of fit with economic misery at 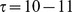 (Pearson's 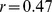 for either), which again is better than the best value for a simple lag (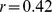 at a lag of 7 years), and is much better for the period 1929 to 2000 (best fit at 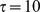 years (Pearson's 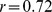).

We also performed the same mood scoring process using different taxonomies of emotional words taken from the Linguistic Inquiry and Word Count (henceforth LIWC), for which the vocabularies of emotions have been evaluated word-by-word by human judges [23]. In particular, we consider the LIWC categories of general affect (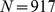 terms), anger (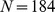), anxiety (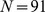), negative emotions (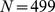), positive emotions (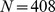) and sadness (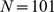). Unlike WNA, LIWC already includes the stems of words together with complete (non-stemmed) words in all of its vocabularies. The analysis was performed as described for WNA (in a non-Porter-stemmed version of the Ngram corpus) and we calculated the literary misery index, 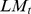, as the difference between the 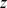-score equivalents in year 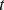 for the 101 listed ‘sadness’ words and for the 408 listed ‘positive emotions’ words. All the analyses were performed on the main corpus (i.e. all books in English). For the 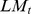 trends derived from WNA, which are the main focus of our analysis, we used additionally the other corpora (fiction books in English, American English books, and British English books).

The last method applied to English was to calculate the literary misery index (“hedonometer”) is described in details in [47]. In this case we extracted from the Ngram corpus the data for 3,686 words which were previously evaluated for their ‘happiness content’ using Amazon's Mechanical Turk. Their normalised frequencies were then weighted with the values provided in [47]. To compare those results with the previous analysis, we 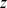-scored them and we considered the opposite of the original index (since we are interested in literary ‘misery’). This method is conceptually different from WNA and LIWC, because it does not consider strictly mood–words; additional terms that are assigned positive or negative feeling, such as ‘food’ (positive) or ‘funeral’ (negative).

To check the generalisation of the results, we also analysed books written in German. The LIWC also allows an analysis of German text as it provides the same emotional categories in German language [69]. To compute a German literary misery index, we used the German corpus of Google Books, which includes approximately 660,000 books, and the analogous LIWC categories of positive emotions (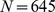) and sadness (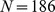). We followed the same methodology as for English books, and for consistency word counts were normalized using the aggregate count of ‘die’,‘der’,‘das’ (feminine, masculine, and neuter versions of ‘the’).

Finally, the economic misery index was calculated by adding the annual historical unemployment rate and annual inflation rate. The data used to construct the misery induces for the different countries were compiled by Ormerod et al. [42] from multiple sources, e.g. [70]–[73].
