# Correction: Books Average Previous Decade of Economic Misery

**DOI:** 10.1371/annotation/83555db1-d407-44c7-82eb-ad2ef10be7f1

**Published:** 2014-01-13

**Authors:** R. Alexander Bentley, Alberto Acerbi, Paul Ormerod, Vasileios Lampos

An affiliation for the last author is incorrectly omitted. Vasileios Lampos is not only affiliated with institution number 4, but also the following institution: Department of Computer Science, University College London, London, United Kingdom.

