# Correction: Books Average Previous Decade of Economic Misery

**DOI:** 10.1371/journal.pone.0099737

**Published:** 2014-06-02

**Authors:** The PLOS ONE Staff

The references to “white circles” and “red circles” within the legends of Figure 1, Figure 2, and Figure 4 are reversed. The “white circles” describe the economic misery index and the “red circles” describe the literary misery curve. Please view Figure 1, Figure 2, and Figure 4 and their corrected legends here.

**Figure 1:**
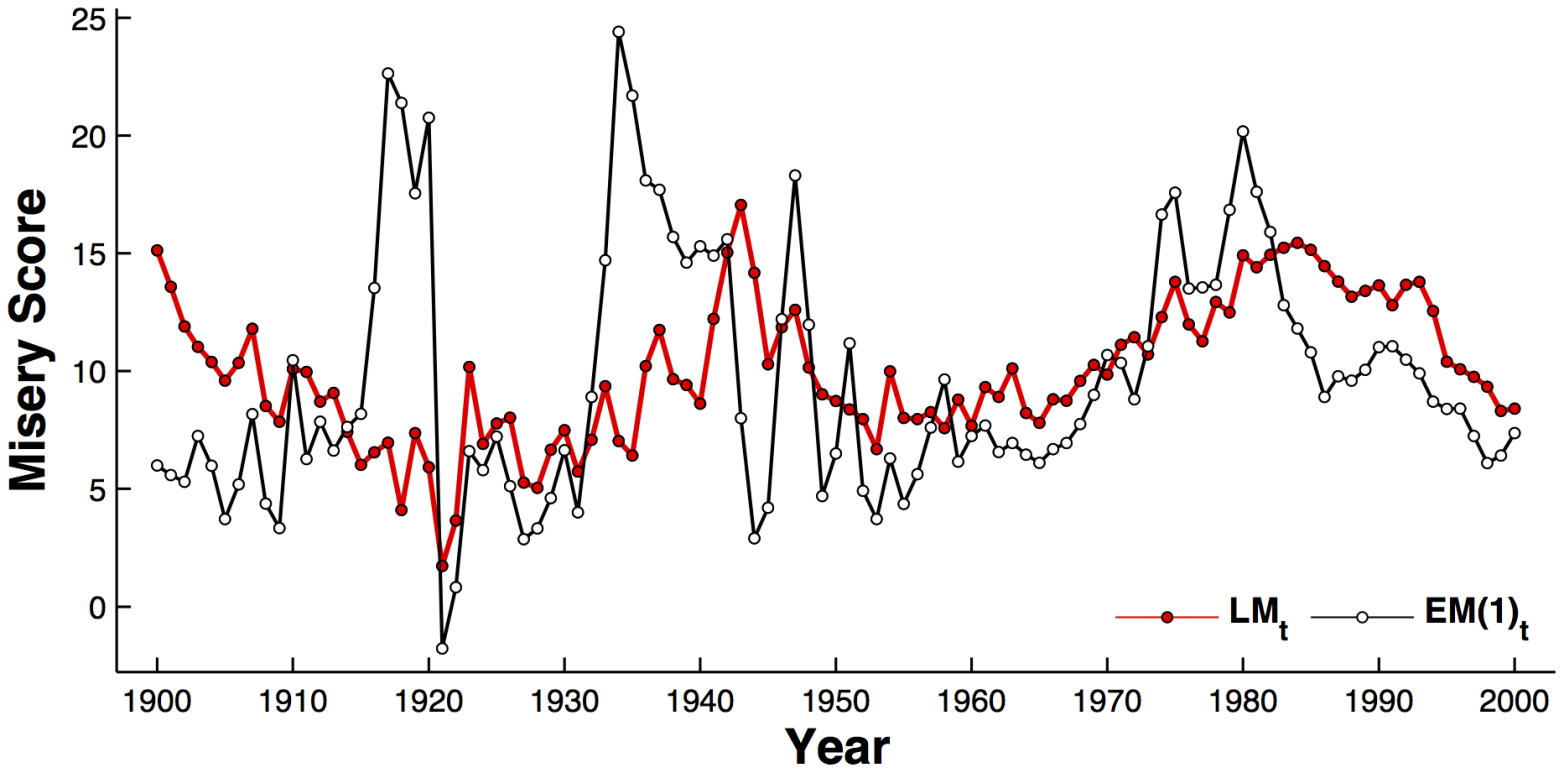
Time series of the literary misery index 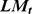 for all books calculated through WNA (red circles), versus the U.S. economic misery index 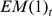 (white circles). 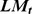
 has been scaled by a factor of 10 to allow a better comparison.

**Figure 2:**
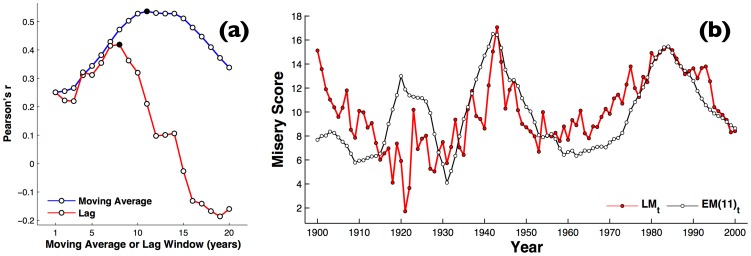
Moving average of economic misery. (a) The effect of varying the moving average period, *τ*, versus a simple lag, on correlation expressed as Pearson's *r* between the time series of 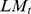 for all books of 1900–2000 and U.S. misery index (b) Time series for 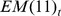, the 11-year moving average of the U.S. misery index (white circles), versus literary misery index, 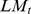, derived from all books calculated through WNA (red circles). Similarly to Figure 1, 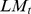 has been scaled by a factor of 10 to allow a better comparison.

**Figure 4:**
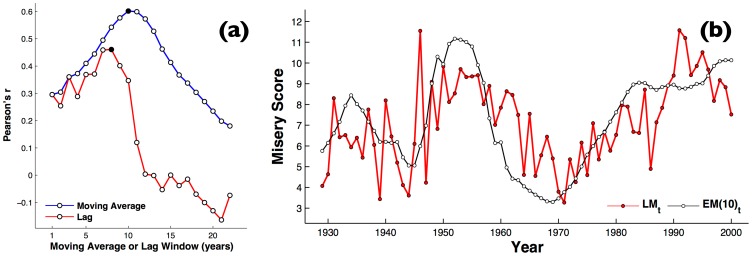
Repeating the analysis on German economic and literary misery. (a) The effect of varying the moving average period, 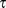, versus as a simple lag, on correlation between for German LIWC and German misery index (b) Time series of the literary misery index for all books calculated through German LIWC (red circles), versus the German economic misery index (white circles).
